# Identification of conserved motifs in the Westnile virus envelope essential for particle secretion

**DOI:** 10.1186/1471-2180-13-197

**Published:** 2013-09-04

**Authors:** Himanshu Garg, Raphael TC Lee, Ng Oon Tek, Sebastian Maurer-Stroh, Anjali Joshi

**Affiliations:** 1 Department of Biomedical Sciences, Texas Tech University Health Sciences Center, 5001 El Paso Dr, MSB-1 Annex, El Paso, TX 79905, USA; 2 Bioinformatics Institute, Agency for Science, Technology and Research, Singapore, Singapore; 3 Department of Infectious Disease, Tan Tock Seng Hospital, Singapore, Singapore; 4 School of Biological Sciences, Nanyang Technological University, Singapore, Singapore

**Keywords:** Flavivirus, WNV, HIV, Virus assembly, Late domains, Alix, Tsg101

## Abstract

**Background:**

Enveloped viruses utilize cellular membranes to bud from infected cells. The process of virion assembly and budding is often facilitated by the presence of certain conserved motifs within viral proteins in conjunction with cellular factors. We hence examined the West Nile Virus (WNV) Envelope protein for the presence of any such motifs and their functional characterization.

**Results:**

We identified conserved ^461^PXAP^464^ and ^349^YCYL^352^ motifs in the WNV envelope glycoprotein bearing resemblance to retroviral late domains. Disruptive mutations of PXAP to LAAL and of the highly conserved Cys^350^ in the YCYL motif, led to a severe reduction in WNV particle production. Similar motifs in case of retroviruses are known to interact with components of host sorting machinery like PXAP with Tsg101 and YXXL with Alix. However, in the case of WNV, siRNA mediated depletion of Alix or Tsg101 did not have an effect on WNV release. Molecular modeling suggested that while the ^461^PXAP^464^ motif is surface accessible and could potentially interact with cellular proteins required for WNV assembly, the ^349^YCYL^352^ motif was found to be internal with Cys^350^ important for protein folding via disulphide bonding.

**Conclusions:**

The conserved ^461^PXAP^464^ and ^349^YCYL^352^ motifs in the WNV envelope are indispensable for WNV particle production. Although these motifs bear sequence similarity to retroviral late domains and are essential for WNV assembly, they are functionally distinct suggesting that they are not the typical late domain like motifs of retroviruses and may play a role other than Alix/Tsg101 utilization/dependence.

## Background

West Nile Virus (WNV) is a single stranded positive sense RNA virus of the genus Flavivirus. The 11Kb RNA genome is translated in the cytoplasm as a polyprotein and processed to yield 3 structural (Capsid C, Premembrane prM/membrane M and Envelope E) and seven non-structural (NS1, NS2A, NS2B, NS3, NS4A, NS4B and NS5) proteins [1]. Co-expression of prM and E proteins alone is sufficient for production of recombinant VLPs [2] that are similar to infectious virions in antigenic properties and have been commonly used to study virus assembly and budding. Although the field of Flavivirus assembly and release remains in its infancy, recent reports have identified certain residues in the prM that are important for WNV particle secretion [3,4]. It is known that WNV genome replication occurs in the cytoplasm in the perinuclear region and virus particles assemble and bud into the Endoplasmic Reticulum (ER) lumen. Subsequently virions are transported to the plasma membrane (PM) via the cellular secretory pathway to be released from cells by exocytosis [5-8].

Following the synthesis of viral genome and proteins, enveloped viruses utilize cellular membranes to bud from infected cells. This is often facilitated by the presence of certain conserved motifs within viral proteins and their ability to interact with the cellular processes/machinery. The best known example of this process is the interaction of retroviral late domain motifs with components of the ESCRT (Endosomal Sorting Complex Required for Transport) sorting machinery to promote budding. Three types of consensus late domain motifs have been identified thus far: (i) the PT/SAP motif recruits the ESCRT-1 component Tsg101 (Tumor Susceptibility growth factor 101) [9,10], (ii) the YXXL late domain motif interacts with the ESCRT associated protein Alix [11,12] and the (iii) PPXY late domain motif binds to the Nedd4 family of E3 ubiquitin ligases that are involved in cargo recruitment during Multivesicular Body (MVB) formation [13,14]. Besides retroviruses, late domain motifs have also been identified in other enveloped viruses like rhabdoviruses (vesicular stomatitis virus, rabies virus) [15-17], filoviruses (ebola, marburg) [18-22], arenaviruses (lymphocytic choriomeningitis virus, lassa virus) [23,24], paramyxoviruses (Nipah virus, Sendai virus) [25,26] and DNA viruses like hepatitis B virus, vaccinia virus, herpes simplex virus-1 and Epstein Barr virus [27-33]. Amongst flaviviruses, the NS3 of Japanese encephalitis virus (JEV) has been shown to associate with Tsg101 [34] while the yellow fever virus (YFV) NS3 has been shown to interact with Alix [35] assisting in virus release. However, currently there is no information on the presence of late domains in WNV proteins.

The process of WNV budding into the lumen of the ER is topologically similar to the process of MVB biogenesis in that both occur in a direction that is away from the cytosol. MVB biogenesis is mediated by the family of ESCRT proteins namely ESCRT-0, -I, -II and -III and other associated proteins like Alix/AIP1. The membrane associated ESCRT-III complexes are finally disassembled and recycled by the ATPase Vps4. A number of enveloped viruses via the conserved late (L) domain motifs that mimic similar motifs in cellular proteins are able to recruit the ESCRT machinery to the site of virus budding [36]. Disruption of L domain motifs or their function leads to defects in the final (late) stages of virus budding characterized by the tethering of virions to the cell surface [9,14,36,37]. Most data on the role of ESCRT proteins and viral late domain motifs has come from research on retroviruses that primarily bud from the plasma membrane. Although there are reports that NS3 of other Flaviviruses can interact with ESCRT components [34,35] there are no such reports for WNV. Furthermore, it is not known whether any late domain like motifs are present in WNV structural proteins especially E protein that is essential for assembly into virus like particles [38].

## Results and discussion

### Identification of conserved motifs in the WNV E protein

**Figure 1:**
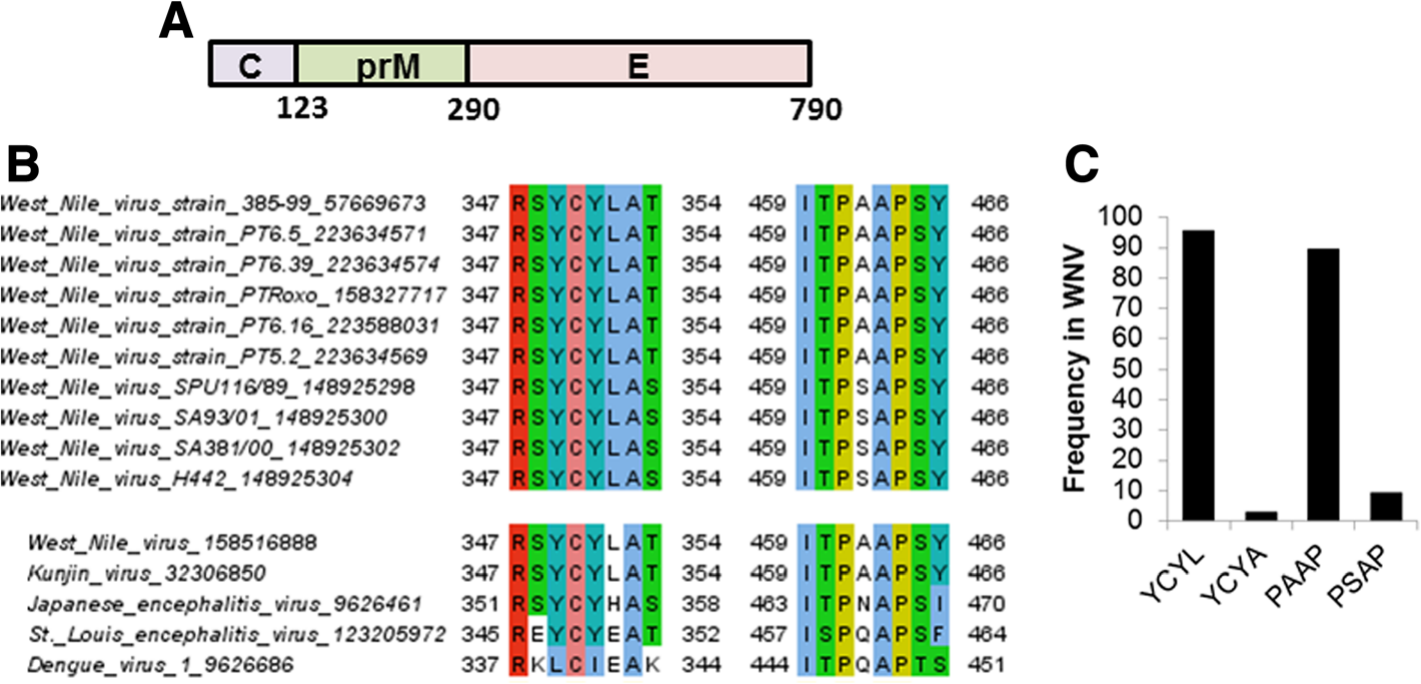
**Sequence analysis of Flavivirus Envelope proteins. (A)** Outline of WNV structural proteins C, PrM and E. **(B)** Presence of conserved ^461^PS/AAP^464^ and ^349^YCYL^352^ motifs in the Flavivirus envelope protein. Selected Flavivirus proteins were downloaded from NCBI [42], aligned with MAFFT [43] and the respective motif regions visualized in Jalview [44] using ClustalX-like coloring based on physicochemical properties and conservation. Virus names are shown left with NCBI GI number. **(C)** Frequency of YCYL and PAAP motif variants in WNV envelope. Significant protein hits (E<0.001) were first identified with Delta-BLAST [45] starting with the sequence of the envelope glycoprotein structure (PDB:2hg0) against NCBI’s non-redundant protein database restricting to West Nile virus sequences only. All hits were next aligned with MAFFT after discarding those without sequence information for the YCYL or PAAP region and removing 100% identical sequences using Jalview. The resulting set of 286 WNV sequences was analyzed for the respective motif occurrences.

In case of Flaviviruses, the structural E protein is necessary for virus assembly and release and the production of recombinant VLPs. Hence, using sequence analysis and information based on work with other viruses we undertook this study to identify the presence of conserved motifs (a vital indicator of the functional importance) in the Flavivirus structural E proteins and determine whether they play a role in virus assembly and release. Sequence analysis of different Flavivirus structural proteins and different WNV isolates revealed the presence of conserved ^461^PXAP^464^ and ^349^YCYL^352^ motifs in the E protein (Figure 1A and B). Similar but less conserved motifs were also present in other Flaviviruses like Kunjin, JEV and St. Louis Encephalitis virus (SLE) (Figure 1B). Analysis of systematically selected WNV E protein sequences suggested that the PAAP motif was present in about 90% of the analyzed sequences while the frequency of the PSAP motif was less than 10% (Figure 1C). The YCYL motif was present in more than 95% of the WNV sequences analyzed. Table 1, depicts the occurrences of the PXAP and YCYL motifs in the protein non-redundant database (nr) database. As expected, sequence motifs that serve some biological functions, occur more often than by chance [39,40] although it deserves mention that these motifs are maintained within the Flavivirus E proteins that themselves are highly conserved. While sequence analysis revealed the predominance of PAAP motifs over PSAP it is unclear as to what advantage the PSAP motif would render in case of WNV. From studies in HIV and that of host proteins like Hrs (Hepatocyte growth factor Receptor Substrate) it is well known that the PSAP motif is a strong binding partner of Tsg101 [41].

**Table 1:** Occurrences of the PXAP and YCYL motifs in the protein nr database

**Motif**	**Actual # occurrences**	**Actual frequency**	Expected # occurrences*	Expected frequency*
**PXAP**	2802870	3.05e-04	1867974	2.03e-04
**YCYL**	11945	1.30e-06	10851	1.18e-06

26,682,258 protein sequences in the non-redundant (nr) protein database downloaded from NCBI on 1st July 2013, were searched for presence of the PXAP and YCYL motifs. The relative abundance of each of the relevant amino acids in the nr database was used to calculate the expected occurrences of the motifs by chance.

*The expected occurrences and frequency were based on the relative abundance of each of the 20 amino acid residues in the nr database. The expected occurrence and frequency of PXAP motif is based on the relative abundance of Proline and Alanine, while that of the YCYL motif is based on the relative abundance of Tyrosine, Cysteine and Leucine in the protein nr database.

### Development of a rapid assay to study WNV assembly and release

**Figure 2:**
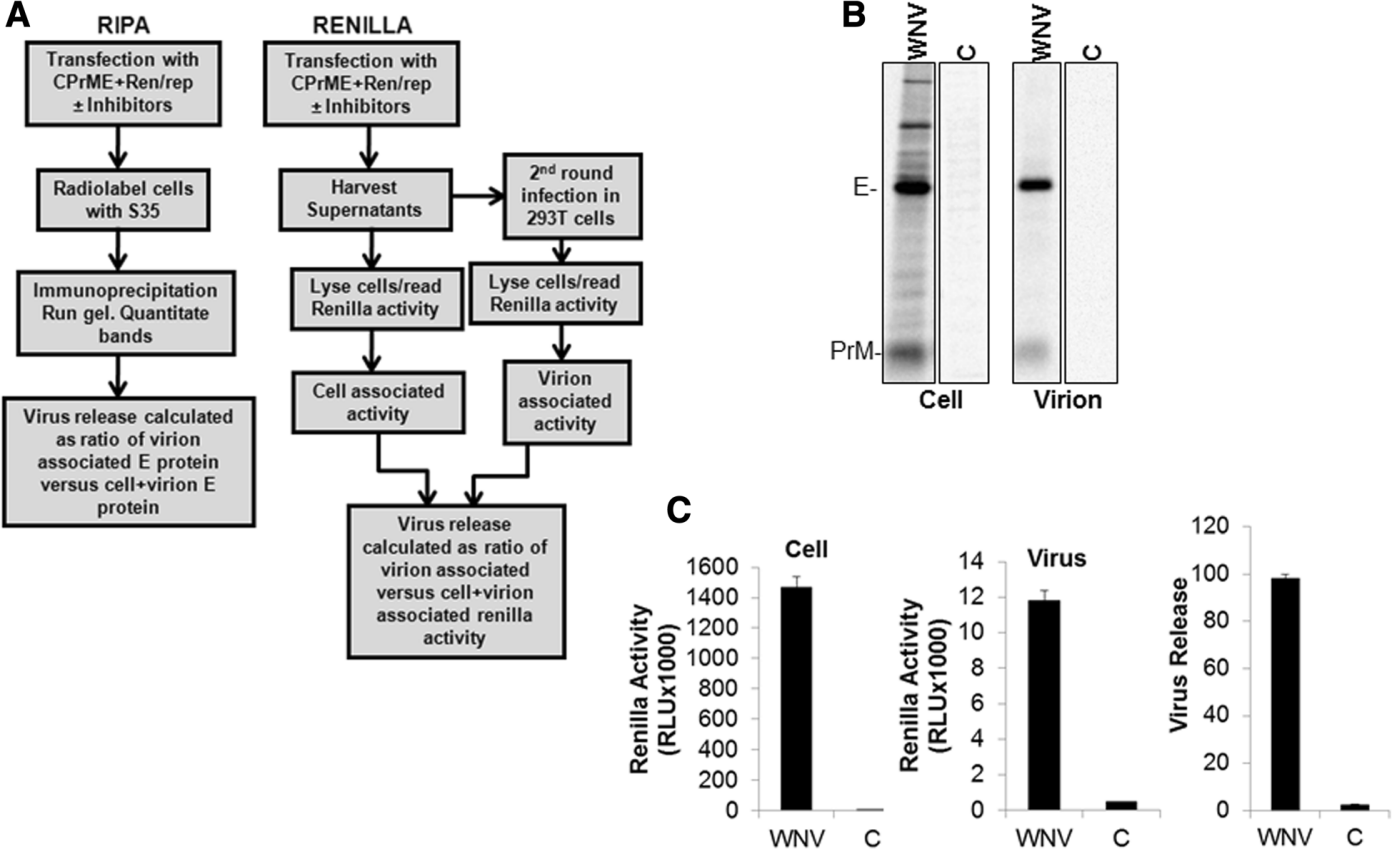
**Rapid assay for studying WNV assembly and release. (A)** Schematic diagram delineating the steps for the rapid Ren-luc based virus release assay and comparing it to the classical radioimmunoprecipitation assay. 293T cells were transfected with WNV-CPrME along with the Ren/Rep plasmids at a ratio of 1:1 or with the pUC vector as control. **(B)** For radioimmunoprecipitation based assay, cells were metabolically labeled with [^35^S]Met-Cyst protein labeling mix (PerkinElmer) in RPMI 1640 medium supplemented with 10% FBS but devoid of Met and Cys 24 h post transfection. Following ultracentrifugation, cell and virus lysates were immunoprecipitated using anti-WNV serum, run on an SDS PAGE gel followed by fluorography. Virus release was calculated as ratio of virion associated versus cell+virion associated E protein. **(C)** For ren-luc based virus release assay, culture supernatants were harvested 24 h post transfection and cells lysed and read for ren-luc activity (cell associated) using the Dual Glo luciferase assay substrate (Promega). Equal volume of the harvested supernatants were then used to infect 293T cells, cells lysed and read for luciferase activity (virion-associated) 24 h post infection. Virus release was calculated as ratio of virion associated versus cell+virion associated ren-luc activity.

We next aimed towards conducting a functional analysis to determine if WNV may utilize the above conserved motifs for virus assembly and release. To this end we developed a rapid renilla luciferase (ren-luc) based virus release assay and compared it to the classical radioimmunoprecipitation based assay (Figure 2). This would not only be a useful tool for rapid siRNA based screens or to identify potential drugs/compounds that inhibit WNV particle production but also obviate the requirement for a BSL3 facility that is necessary for working with infectious WNV. 293T cells were transfected with CprME and WNV Ren/Rep plasmids [46]. Culture supernatants were harvested 24 h post transfection and cells lysed and read for ren-luc activity (cell associated, Figure 2A and C) using the Dual Glo luciferase assay substrate (Promega). Equal volume of the harvested supernatants were then used to infect 293T cells, cells lysed and read for luciferase activity (virion-associated) 24 h post infection (Figure 2A and C). Virus release was calculated as ratio of virion associated ren-luc/(cell+virion associated ren-luc) activity. In parallel, classical radioimmunoprecipitation based virus release assay [47] was also conducted to determine the validity of the rapid assay described above (Figure 2A and B). Although, the luciferase based rapid assay also accounts for entry defects in virions, it is a convenient high throughput method for identification of general inhibitors of the virus life cycle.

### Tsg-5’ and Alix-V domain expression inhibits WNV assembly and release

**Figure 3:**
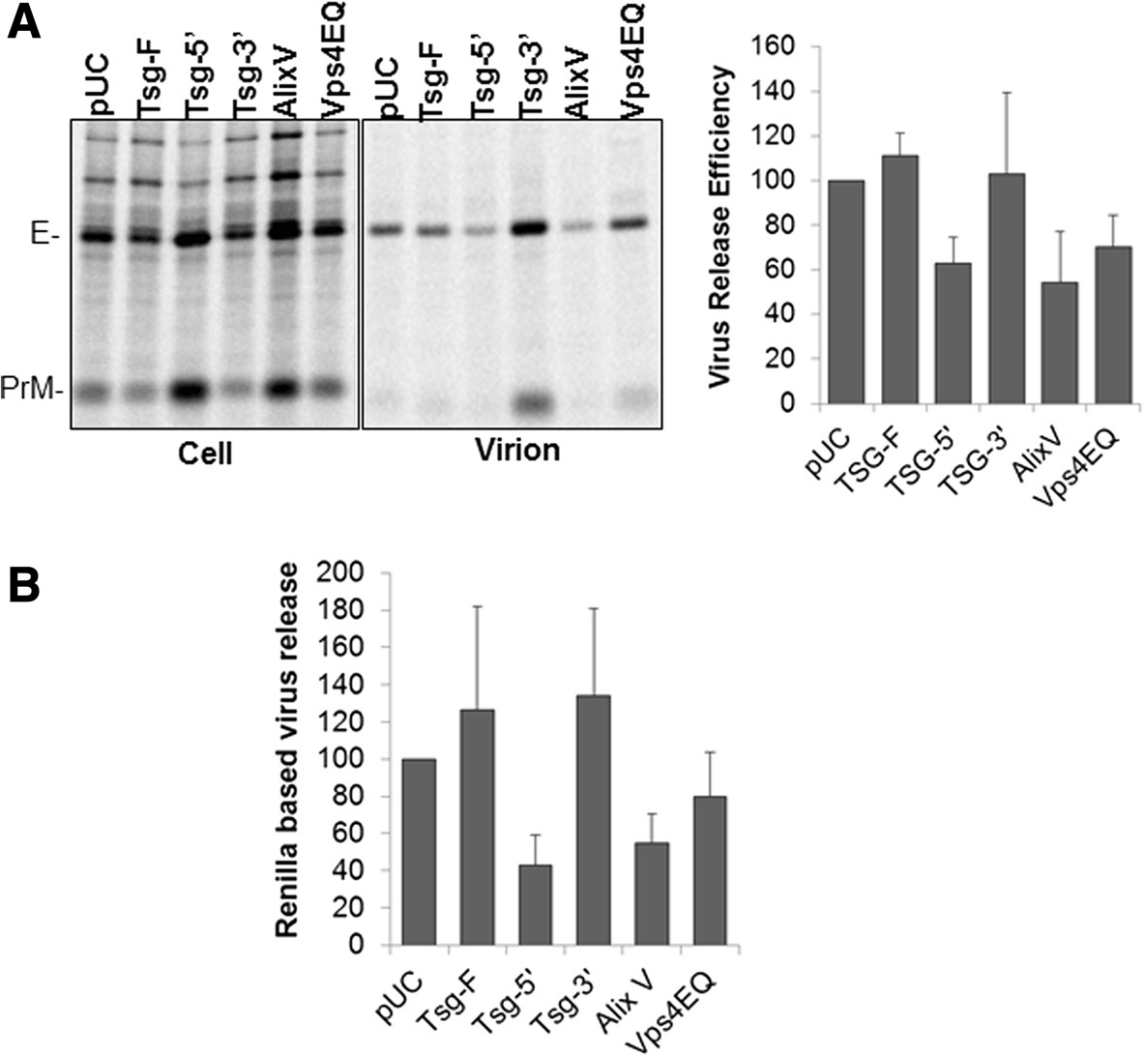
**WNV release is inhibited on expression of Tsg-5’ and Alix V domain.** 293T cells were transfected with WNV-CPrME and Ren/Rep plasmids along with control pUC or the indicated cellular protein expression constructs. Virus release was determined using the **(A)** classical radioimmunoprecipitation technique and **(B)** the rapid ren-luc based assay. Data represent mean ± SD from 3 **(A)** or 4 **(B)** independent experiments.

As mentioned above, the conserved motifs identified in the WNV envelope resembled the late domain like motifs of retroviruses. We hence asked whether some of the well characterized inhibitors of ESCRT pathway previously used to study retrovirus budding would affect WNV assembly and release. To inhibit Tsg101 we utilized either Tsg-5’ expression vector that prevents HIV Gag-Tsg101 interaction or Tsg-F and TSG-3’ that have been shown to inhibit HIV release by globally disrupting the endosomal sorting machinery [48,49]. We also used a transdominant form of Vps4 (Vps4EQ) that prevents the dissociation of ESCRT-III components at the endosomal membrane thereby inhibiting HIV-1 and Murine Leukemia Virus (MLV) budding [49-51], [52]. Similarly, the V domain of Alix (residues 364–716) which is known to bind both Equine Infectious Anemia Virus (EIAV) and HIV-1 Gag acting as a dominant-negative inhibitor of virus release [51,53,54] was also used. 293T cells were transfected to express CprME, WNV Ren/Rep plasmids in the presence of either control plasmid (pUC) or Tsg-F, Tsg-5’ , Tsg-3’ [49], Alix-V [53] or Vps4EQ [50] expression vectors. Virus release efficiency was then calculated by both the rapid assay and classical virus release assay. Interestingly, the expression of Tsg-5’ and Alix-V domain modestly diminished WNV release whereas no significant effect on virus release was observed on expression of Tsg-3’ Tsg-F or Vps4EQ (Figure 3A and B). While it is known that expression of Tsg-5’ affects HIV-1 release by affecting late domain function [48,49], the precise mechanism via which Tsg-3’ , Tsg-F or Alix-V domain affect HIV release remains unknown. They could either be affecting the function of specific host proteins or universally disrupting the cell sorting machinery utilized for WNV particle production.

### Mutations of the conserved PAAP and YCYL motifs in WNV envelope inhibits virus particle production

**Figure 4:**
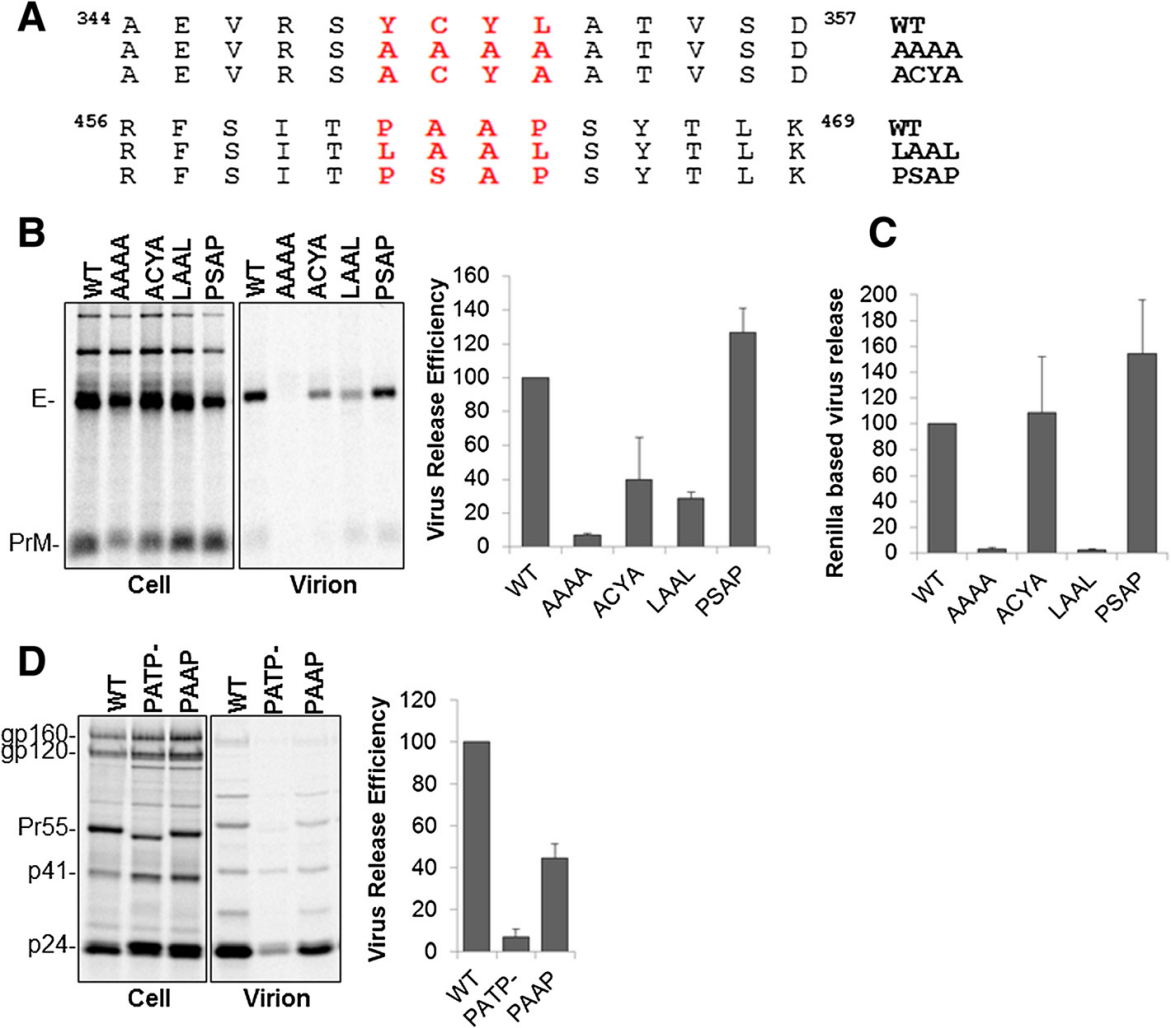
**Mutation of PAAP motif to LAAL significantly diminishes WNV release. (A)** Sequence of the ^461^PS/AAP^464^ and ^349^YCYL^352^ motif bearing region and their mutagenesis strategy. 293T cells were transfected with WNV-CPrME WT or the indicated mutant DNAs along with the Ren/Rep plasmid. Virus release was determined using the **(B)** classical radioimmunoprecipitation technique and **(C)** the rapid ren-luc based assay. Pooled data (mean ± SD) from 3 **(A)** or 4 **(B)** independent experiments is shown. **(D)** HIV-PAAP mutant is capable of efficient release when compared to the PTAP minus mutant. 293T cells were transfected with HIV pNL4-3 WT, PTAP- or PAAP DNA. Virus release was determined 24 h post transfection after radiolabeling and immunoprecipitation with HIV-Ig.

To further examine the relevance of these conserved PXAP and YCYL motifs in WNV assembly and release, we constructed mutations in the PAAP residues to either LAAL or PSAP (Figure 4A) using site directed mutagenesis. Interestingly, mutation of PAAP to LAAL caused a severe defect in virus budding, while mutation of the residues to PSAP led to virus release efficiency that was modestly better than WT (Figure 4B and C). We also mutated the YCYL domain to ACYA or AAAA. Interestingly, mutation of the above motifs to AAAA but not ACYA caused a severe defect in virus release (Figure 4B and C). It is worth mentioning that disruptive mutations to AAAA may also have other adverse side effects since the highly conserved cysteine residue may be involved in maintaining protein structure and stability.

It has previously been shown in context of HIV-1 that the PAAP motif interacts poorly with Tsg101 in in-vitro binding assays using purified proteins [9,21,55]. Since a large number of WNV isolates naturally bear a PAAP motif at position 461–464 instead of PTAP, we wanted to determine if a PAAP motif in the HIV p6 would permit virus release. We hence mutated the PTAP motif in HIV to PAAP and determined virus release. Although HIV-PAAP was released less efficiently than WT-HIV, it was significantly better than the PTAP deleted mutant (Figure 4D). These findings, at least in case of HIV where disruption of PT/SAP Tsg101 interaction significantly affects virus release are indicative that the PAAP motif may still be capable of binding Tsg101 albeit at a lower efficiency. Thus a PAAP motif can act as a functional late domain for HIV and hence could do the same for WNV isolates that predominantly bear PAAP motifs. Our findings are consistent with those of Demirov et al. [56] although the possibility that the PAAP motif is capable of interacting directly or indirectly with certain other host factors that favor HIV and/or WNV release cannot be ruled out.

### Depletion of endogenous Alix or Tsg101 does not inhibit WNV assembly and release

**Figure 5:**
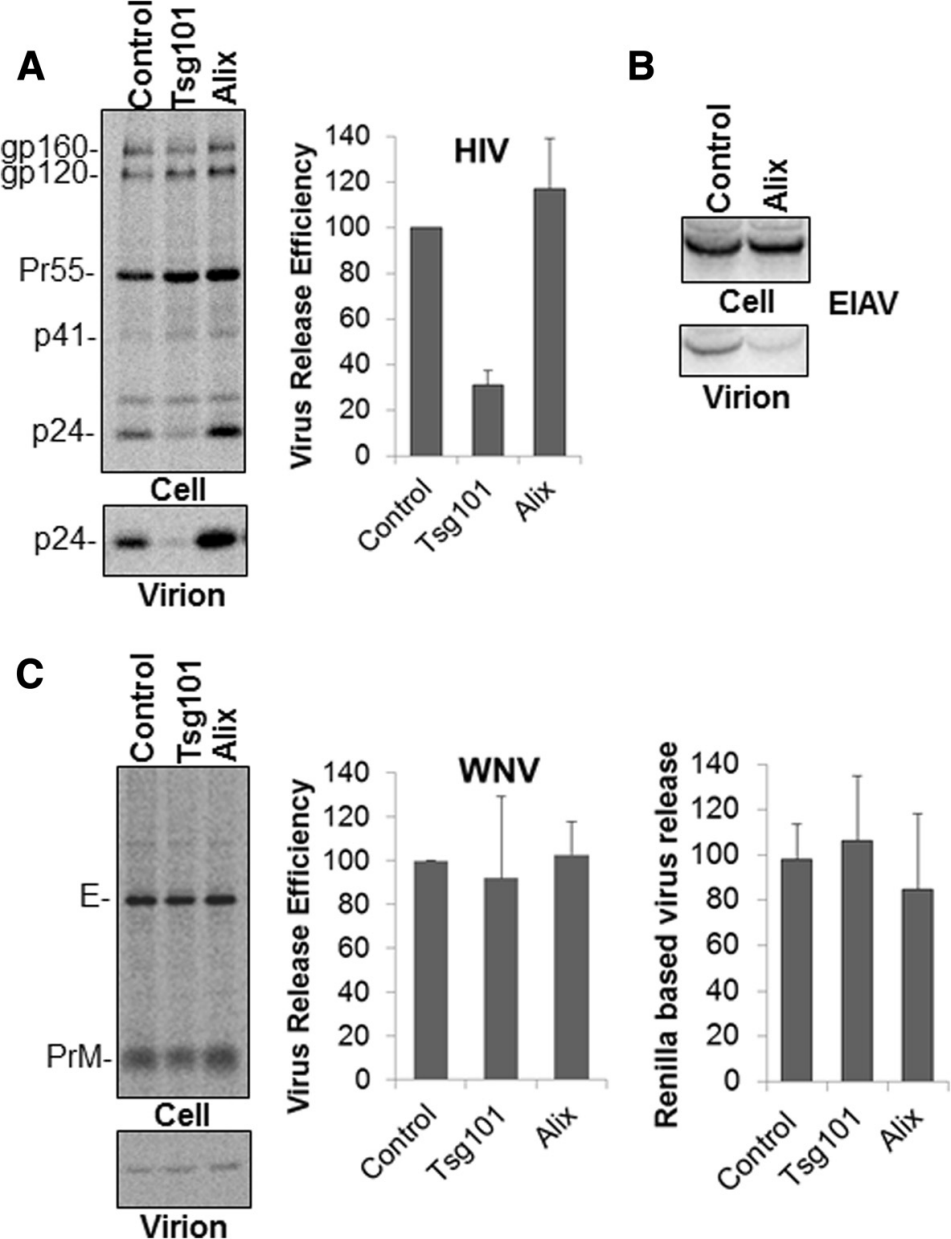
**Depletion of endogenous Tsg101 or Alix using specific siRNA does not inhibit WNV release.** 293T cells were transfected with control, Alix or Tsg101 siRNA. 24 h post transfection cells were transfected again with respective siRNAs along with **(A)** WT HIV-1 pNL4-3 DNA **(B)** WT EIAV Gag DNA or **(C)** WNV-CPrME plus the Ren/Rep plasmids. Virus release was determined after radiolabeling and immunoprecipitation for HIV and WNV, via western blotting for EIAV and also by the rapid ren-luc based assay for WNV. Data represent mean ± SD from 3 independent experiments **(A**&**C)**. For the ren-luc based WNV assay one representative of 3 independent experiments is shown.

Our findings that Tsg-5’ expression inhibits WNV release suggests a role for the ESCRT pathway in WNV budding. However, in other enveloped viruses that bear late domains (e.g. Gag of retroviruses, matrix of rhabdoviruses, VP40 of Ebolavirus) these motifs are located on the cytoplasmic side of the membrane and thus would be able to interact with ESCRT proteins to facilitate budding and particle release. The Flavivirus E protein on the other hand is translated into the lumen of the ER and hence these conserved motifs in WNV E protein would only be minimally exposed to the cytoplasmic side of intracellular vesicles or the plasma membrane. Hence in order to confirm the role of Tsg101 and/or Alix in WNV assembly and release we used a siRNA based approach. For this 293T cells were knocked down of endogenous Tsg101 and Alix expression using specific siRNAs and WNV release determined using both Ren-luc based and immunoprecipitation based virus release assays. As shown in Figure 5A and C, while Tsg101 depletion had no effect on WNV particle secretion, as expected, it caused a severe reduction in HIV-1 release. Alix depletion on the other hand had no effect on either HIV or WNV release (Figure 5A and C) but diminished EIAV release (Figure 5B). Thus while the conserved PXAP and YCYL motifs in WNV are important for virus assembly and release, it is most likely not due to dependence on the ESCRT component Tsg101 or the associated factor, Alix.

### In the WNV E protein, the PAAP motif is surface located while the YCYL motif is deeply buried

**Figure 6:**
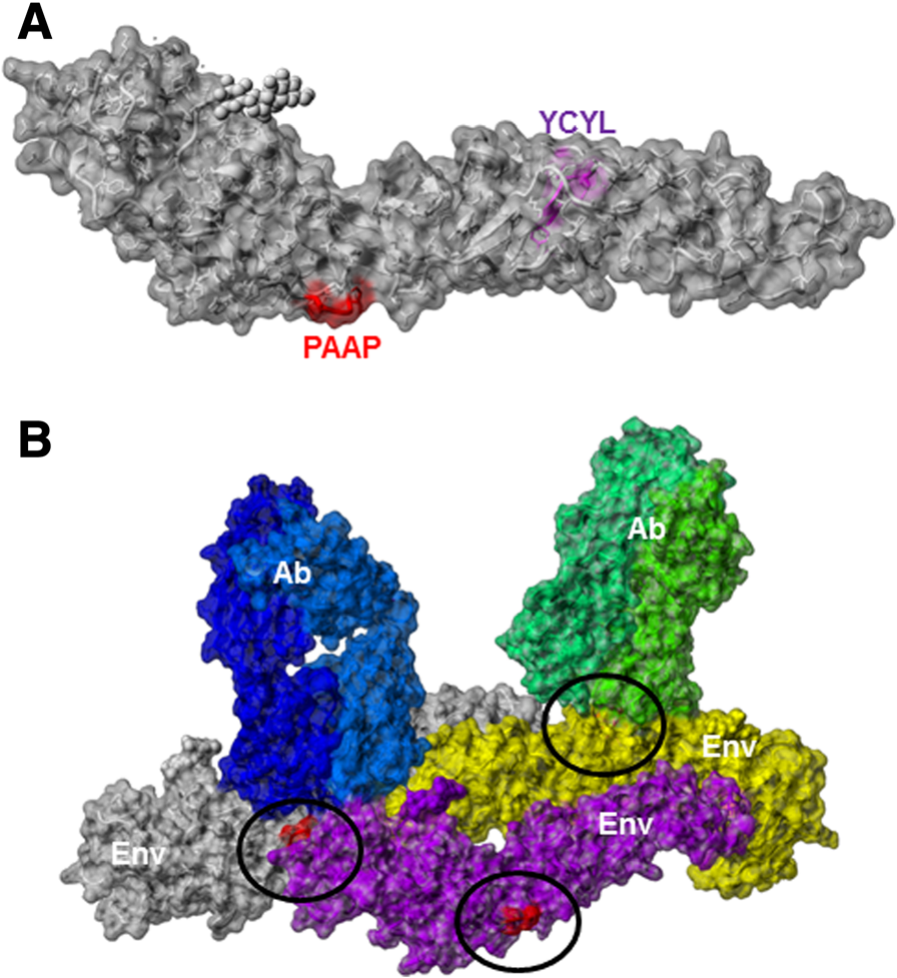
**Crystal structure of West Nile virus envelope glycoprotein visualized with Yasara **[57]. **(A)** Analyzed motifs on PDB:2hg0 [58] highlighted in red (PAAP) or magenta (YCYL). Structural analysis suggests that the PAAP motif is surface accessible while the YCYL motif is buried. **(B)** Analysis of the envelope protein in context of the assembled viral envelope PDB:3iyw [59]. Three envelope proteins are shown in gray, purple and yellow. The PAAP surface motif (red residues in black circles) appears to form part of the interface between the envelope subunits. It also lies adjacent to the discontinuous epitope recognition site of co-crystallized neutralizing antibodies (blue and green).

Our siRNA mediated depletion studies above suggested that WNV may not rely on the ESCRT host cell sorting machinery for assembly and release. Thus, it is plausible that these motifs may interact with other host factors to facilitate the assembly of the virion particles. In fact our structural analysis shows that the PXAP motif is surface accessible and could participate in protein interactions with yet unidentified cellular factors (Figure 6A). In the context of the viral capsid made up of multiple envelope (E) proteins the PXAP surface motif appears to form part of the interface between the envelope subunits (Figure 6B). It also lies adjacent to the discontinuous epitope recognition site of co-crystallized neutralizing antibodies. On the other hand the YCYL motif is deeply buried and forms part of the structural core with the central cysteine participating in formation of a critical disulfide bridge (Figure 6A). This is in agreement with our findings where mutation of the YCYL motif to ACYA had little effect on virus release but mutation to AAAA severely affected budding possibly via loss of the disulphide bridging cysteine.

## Conclusions

In this study, we have developed a rapid assay to study WNV assembly and release and identified conserved motifs in the viral envelope (E) that have functional relevance. These motifs bear sequence homology to late domain like motifs described in retroviruses. Experiments aimed at elucidating their role demonstrated that while expression of Tsg-5’ and Alix-V domain modestly inhibited WNV particle production, expression of Vps4EQ had no effect on WNV release. These data combined with the fact that siRNA mediated depletion of Alix or Tsg101 did not affect WNV release argues against their utilization or the ESCRT pathway by WNV. For instance, it has been documented that HSV possesses PT/SAP and YXXL motifs in several of its proteins but virus particle production is independent of Alix or Tsg101 expression [60]. Likewise, the PSAP motifs are conserved amongst the Vesiculovirus M protein without possessing L domain activity [61,62]. However, the conserved nature of these domains in WNV and reduced virus release upon disruptive mutations argues in favor of a role in virus assembly via yet unidentified mechanism/s.

Our data are also reminiscent of the effects of Alix V domain expression versus Alix depletion on HIV particle production. While siRNA depletion of Alix does not affect HIV release, dominant negative inhibition via Alix V domain expression does [11,53]. Moreover, it was recently demonstrated that the Alix V domain is capable of interacting with ubiquitin [51,63,64]. It is also known that ubiquitination plays a role in both HIV and flavivirus particle production [65,66]. It is thus plausible that expression of the Alix V domain may alter ubiquitin dependent cellular functions thereby affecting WNV particle production. The precise mechanism behind this phenomenon with respect to HIV-1 remains to be elucidated. The fact that some WNV strains like Sarafend exhibits significant budding from the plasma membrane [67] would favor a role of ESCRT components like Alix and Tsg101 for budding.

Sequence analysis and information based on other viruses showed the presence of PXAP and YXXL conserved motifs in the E protein of Flaviviruses and different WNV strains, motifs that resemble the retroviral late domain-like motifs. It is worth mentioning that sequence analysis of a large portion of several different Flavivirus E proteins showed only 18% conservation in the amino acid residues, although the number does reflect the maximum diversity across the whole Flavivirus family [68]. This conservation was mostly seen on the inner surface of the monomers plausibly as a result of neutralizing antibody pressure. On the contrary, the PXAP and YCYL motifs were quite conserved indicating their functional relevance. Moreover, substantial changes in the consensus sequences are also found to occur in only a few areas of the E protein and may have relevance to growth in insect cells versus vertebrates [69]. Although mutational analysis confirms the importance of these domains in WNV assembly and particle formation, the role of Tsg101 and Alix in this phenomenon remains inconclusive from this study. Molecular modeling shows that the PXAP domain is present on the surface of the E protein and could potentially interact with cellular factors. On the other hand the YCYL conserved domain consisted of a conserved cysteine that is involved in disulphide bonding and protein folding. Although the YCYL motif may be critical in maintaining structure of the virus, the conservation of this motif and its functional relevance has neither been studied nor demonstrated in other Flaviviruses. Moreover, the same was not true for the PXAP domain. Interestingly, mutation of the PAAP motif to PSAP, which is an optimal binding partner for cellular sorting proteins modestly enhanced virus release. Considering the presence of only PAAP and PSAP at positions 461–464 in all the WNV sequences analyzed, the importance of this domain in virus assembly cannot be ignored. While the cellular sorting partner of PS/AAP domain in WNV could not be identified, our study opens the gate for further investigation into understanding WNV and Flavivirus assembly in general.

Further studies are needed to determine the precise mechanism via which these motifs, specially the PXAP domain, regulates WNV assembly and release and whether it functions via interaction with certain host factors or merely play a structural role in regulating virus assembly and release.

## Methods

### Cell culture and transfections

293T cells were cultured in DMEM supplemented with 10% FBS. All transfections were performed using Lipofactamine2000™ reagent (Invitrogen) as per the manufacturer’s instructions. In cases where transfections involved multiple DNAs, efficiency of co-transfection was carefully controlled by using an equal amount of plasmid expression vectors for each well and adjusting the total input DNA in each well to be constant by using pUC DNA.

### Plasmids, antibodies, cell culture reagents, and siRNAs

The WNV CprME and Ren/Rep plasmids have been described previously [46] and were kindly provided by Dr. Ted Pierson (NIAID). Mutations in the CprME ^461^PAAP^464^ and ^349^YCYL^352^ motifs to PSAP, LAAL, ACYA and AAAA were constructed by site directed mutagenesis (Stratagene) using specific primer pairs. The full-length HIV-1 proviral clone pNL4-3 [70] and its PTAP minus derivative have been described previously [56]. The HIV PAAP mutant in the pNL4-3 backbone was constructed by site directed mutagenesis. Hemagglutinin (HA)-tagged derivatives of Tsg101-TSG-5′ and TSG-3′ in the pcGNM2 expression as well as the full-length Tsg101 expression vector (pcGNM2/TSG-F) have been previously described [49]. pEGFP-C2:VPS4A(E228Q), expressing an ATPase-deficient mutant of VPS4A fused to GFP has been previously described [50]. Expression vectors for the V domain of Alix (pcGNM2/hAlix(364–716) have been described [54]. The EIAV Gag expression vector (pPRE/GagEIAV) has been described [71].

### Metabolic labeling and immunoprecipitation

The protocol for radiolabeling and immunoprecipitation of cell and virus lysates has been described in detail previously [72]. Briefly, transfected cells were starved for 30 min in RPMI medium lacking Met and Cys. Thereafter, cells were incubated for 2–3 h in RPMI medium supplemented with FBS and [^35^S]Met/Cys. Culture supernatants were filtered and subjected to ultracentrifugation at 100,000 x g for 45 min. Cell and virion samples were lysed in cell lysis buffer (0.5% Triton X-100, 300 mM NaCl, 50 mM Tris [pH 7.5] containing protease inhibitors [Complete; Roche]). Thereafter, they were immunoprecipitated either with HIV-Ig (Kindly provided by the NIH AIDS research and reference reagent program) or anti-WNV serum (Kindly provided by Dr. Robert B. Tesh, University of Texas Medical Branch, Galveston) coated Protein A beads. Immunoprecipitated cell lysates were washed three times in RIPA buffer and once with SDS-DOC wash (0.1% sodium dodecyl sulfate, 300 mM NaCl, 50 mM Tris [pH 7.5], 2.5 mM deoxycholic acid), resolved by SDS-PAGE followed by PhosphorImager analysis. Virus release efficiency was calculated as ratio of virion associated versus total cell plus virion associated HIV-1 Gag or WNV E protein.

### Renilla based virus release assay

The overall strategy for this assay is summarized in Figure 2A. 293T cells were transfected with CprME and WNV Ren/Rep plasmids [46]. Culture supernatants were harvested 24 h post transfection and cells lysed and read for ren-luc activity using the Dual Glo luciferase assay substrate (Promega). Equal volume of the harvested supernatants were then used to infect 293T cells, cells lysed and read for luciferase activity (virion-associated) 24 h post infection. Virus release was calculated as ratio of virion associated ren-luc/(cell+virion associated ren-luc) activity. The overall strategy is summarized in Figure 2A.

### Sequence analysis

Selected Flavivirus proteins were downloaded from NCBI [42]. The NCBI database was searched for sequences for complete or almost full length (>3300 amino acids) polyproteins from Flaviviruses and selected the ones with species name including West Nile Virus. If multiple sequences were available per virus name, only the longest sequence was considered. This yielded 11 different West Nile virus sequences with separate strain designations (strain name and GI numbers shown in alignment). The downloaded sequences were aligned with MAFFT [43] and the respective motif regions visualized in Jalview [44] using ClustalX-like coloring based on physicochemical properties and conservation. To systematically count the frequency of YCYL and PAAP motif variants in WNV, we first identified significant protein hits (E<0.001) with Delta-BLAST [45] starting with the sequence of the envelope glycoprotein structure (PDB:2hg0) against NCBI’s non-redundant protein database restricting to West Nile virus sequences only. Next, we aligned all hits with MAFFT [43] and discarded those without sequence information for the YCYL or PAAP region and removed 100% identical sequences using Jalview [44], leaving us with a set of 286 WNV sequences for which we calculated the respective motif occurrences.

The strain designations as listed in the alignment were taken from the NCBI taxonomy on West Nile viruses: http://www.ncbi.nlm.nih.gov/Taxonomy/Browser/wwwtax.cgi?id=11082.

Several of these strains like Sarafend belong to the pathogenic lineage 2. These are: West Nile virus H442, West Nile virus SA381/00, West Nile virus SA93/01, West Nile virus SPU116/89. Please note that the Kunjin virus has been recognized as WNV strain which is also visible by the identical sequences in the 2 displayed patterns.

## Abbreviations

HIV: Human immunodeficiency virus type 1; WNV: West Nile virus; EIAV: Equine infectious anemia virus; JEV: Japanese encephalitis virus; YFV: Yellow fever virus; SLE: St. Louis encephalitis virus; C: Capsid; prM: Pre membrane; NS: Non-structural; VLPs: Virus like particles; ESCRT: Endosomal sorting complex required for transport; Tsg101: Tumor susceptibility growth factor 101; MVB: Multi vesicular bodies; ER: Endoplasmic reticulum.

## Competing interests

The authors declare they have no competing interests.

## Authors’ contributions

HG and AJ designed the study, performed experiments, analyzed data and wrote the manuscript. RL, OT and SM performed sequence analysis, analyzed data and wrote the manuscript. All authors read and approved the final manuscript.

## References

[B1] Brinton MA The molecular biology of West Nile Virus: a new invader of the western hemisphere Annu Rev Microbiol 2002 56 371 402 10.1146/annurev.micro.56.012302.160654 12142476

[B2] Lindenbach BD Thiel HJ Rice CM Flaviviridae: the viruses and their replication 2007 Philadelphia, PA: Fields virology Lippincott William & Wilkins 1101 1152

[B3] Calvert AE Huang CY Blair CD Roehrig JT Mutations in the West Nile prM protein affect VLP and virion secretion in vitro Virology 2012 433 35 44 10.1016/j.virol.2012.07.011 22858174 PMC5812725

[B4] Setoh YX Prow NA Hobson-Peters J Lobigs M Young PR Khromykh AA Hall RA Identification of residues in West Nile virus pre-membrane protein that influence viral particle secretion and virulence J Gen Virol 2012 93 1965 1975 10.1099/vir.0.044453-0 22764317

[B5] Li J Bhuvanakantham R Howe J Ng ML Identifying the region influencing the cis-mode of maturation of West Nile (Sarafend) virus using chimeric infectious clones Biochem Biophys Res Commun 2005 334 714 720 10.1016/j.bbrc.2005.06.150 16018972

[B6] Mackenzie JM Westaway EG Assembly and maturation of the flavivirus Kunjin virus appear to occur in the rough endoplasmic reticulum and along the secretory pathway, respectively J Virol 2001 75 10787 10799 10.1128/JVI.75.22.10787-10799.2001 11602720 PMC114660

[B7] Mason PW Maturation of Japanese encephalitis virus glycoproteins produced by infected mammalian and mosquito cells Virology 1989 169 354 364 10.1016/0042-6822(89)90161-X 2523178 PMC7125691

[B8] Nowak T Farber PM Wengler G Analyses of the terminal sequences of West Nile virus structural proteins and of the in vitro translation of these proteins allow the proposal of a complete scheme of the proteolytic cleavages involved in their synthesis Virology 1989 169 365 376 10.1016/0042-6822(89)90162-1 2705302

[B9] Garrus JE von Schwedler UK Pornillos OW Morham SG Zavitz KH Wang HE Wettstein DA Stray KM Cote M Rich RL Tsg101 and the vacuolar protein sorting pathway are essential for HIV-1 budding Cell 2001 107 55 65 10.1016/S0092-8674(01)00506-2 11595185

[B10] Gottlinger HG Dorfman T Sodroski JG Haseltine WA Effect of mutations affecting the p6 gag protein on human immunodeficiency virus particle release Proc Natl Acad Sci USA 1991 88 3195 3199 10.1073/pnas.88.8.3195 2014240 PMC51412

[B11] Martin-Serrano J Yarovoy A Perez-Caballero D Bieniasz PD Divergent retroviral late-budding domains recruit vacuolar protein sorting factors by using alternative adaptor proteins Proc Natl Acad Sci USA 2003 100 12414 12419 10.1073/pnas.2133846100 14519844 PMC218772

[B12] Strack B Calistri A Craig S Popova E Gottlinger HG AIP1/ALIX is a binding partner for HIV-1 p6 and EIAV p9 functioning in virus budding Cell 2003 114 689 699 10.1016/S0092-8674(03)00653-6 14505569

[B13] Xiang Y Cameron CE Wills JW Leis J Fine mapping and characterization of the Rous sarcoma virus Pr76gag late assembly domain J Virol 1996 70 5695 5700 8764091 10.1128/jvi.70.8.5695-5700.1996 PMC190537

[B14] Freed EO Viral late domains J Virol 2002 76 4679 4687 10.1128/JVI.76.10.4679-4687.2002 11967285 PMC136167

[B15] Craven RC Harty RN Paragas J Palese P Wills JW Late domain function identified in the vesicular stomatitis virus M protein by use of rhabdovirus-retrovirus chimeras J Virol 1999 73 3359 3365 10074190 10.1128/jvi.73.4.3359-3365.1999 PMC104100

[B16] Harty RN Paragas J Sudol M Palese P A proline-rich motif within the matrix protein of vesicular stomatitis virus and rabies virus interacts with WW domains of cellular proteins: implications for viral budding J Virol 1999 73 2921 2929 10074141 10.1128/jvi.73.4.2921-2929.1999 PMC104051

[B17] Jayakar HR Murti KG Whitt MA Mutations in the PPPY motif of vesicular stomatitis virus matrix protein reduce virus budding by inhibiting a late step in virion release J Virol 2000 74 9818 9827 10.1128/JVI.74.21.9818-9827.2000 11024108 PMC102018

[B18] Harty RN Brown ME Wang G Huibregtse J Hayes FP A PPxY motif within the VP40 protein of Ebola virus interacts physically and functionally with a ubiquitin ligase: implications for filovirus budding Proc Natl Acad Sci USA 2000 97 13871 13876 10.1073/pnas.250277297 11095724 PMC17668

[B19] Kolesnikova L Bamberg S Berghofer B Becker S The matrix protein of Marburg virus is transported to the plasma membrane along cellular membranes: exploiting the retrograde late endosomal pathway J Virol 2004 78 2382 2393 10.1128/JVI.78.5.2382-2393.2004 14963134 PMC369247

[B20] Licata JM Simpson-Holley M Wright NT Han Z Paragas J Harty RN Overlapping motifs (PTAP and PPEY) within the Ebola virus VP40 protein function independently as late budding domains: involvement of host proteins TSG101 and VPS-4 J Virol 2003 77 1812 1819 10.1128/JVI.77.3.1812-1819.2003 12525615 PMC140960

[B21] Martin-Serrano J Zang T Bieniasz PD HIV-1 and Ebola virus encode small peptide motifs that recruit Tsg101 to sites of particle assembly to facilitate egress Nat Med 2001 7 1313 1319 10.1038/nm1201-1313 11726971

[B22] Urata S Noda T Kawaoka Y Morikawa S Yokosawa H Yasuda J Interaction of Tsg101 with Marburg virus VP40 depends on the PPPY motif, but not the PT/SAP motif as in the case of Ebola virus, and Tsg101 plays a critical role in the budding of Marburg virus-like particles induced by VP40, NP, and GP J Virol 2007 81 4895 4899 10.1128/JVI.02829-06 17301151 PMC1900181

[B23] Perez M Craven RC de la Torre JC The small RING finger protein Z drives arenavirus budding: implications for antiviral strategies Proc Natl Acad Sci USA 2003 100 12978 12983 10.1073/pnas.2133782100 14563923 PMC240730

[B24] Urata S Noda T Kawaoka Y Yokosawa H Yasuda J Cellular factors required for Lassa virus budding J Virol 2006 80 4191 4195 10.1128/JVI.80.8.4191-4195.2006 16571837 PMC1440458

[B25] Ciancanelli MJ Basler CF Mutation of YMYL in the Nipah virus matrix protein abrogates budding and alters subcellular localization J Virol 2006 80 12070 12078 10.1128/JVI.01743-06 17005661 PMC1676283

[B26] Sakaguchi T Kato A Sugahara F Shimazu Y Inoue M Kiyotani K Nagai Y Yoshida T AIP1/Alix is a binding partner of Sendai virus C protein and facilitates virus budding J Virol 2005 79 8933 8941 10.1128/JVI.79.14.8933-8941.2005 15994787 PMC1168738

[B27] Calistri A Sette P Salata C Cancellotti E Forghieri C Comin A Gottlinger H Campadelli-Fiume G Palu G Parolin C Intracellular trafficking and maturation of herpes simplex virus type 1 gB and virus egress require functional biogenesis of multivesicular bodies J Virol 2007 81 11468 11478 10.1128/JVI.01364-07 17686835 PMC2045546

[B28] Chua HH Lee HH Chang SS Lu CC Yeh TH Hsu TY Cheng TH Cheng JT Chen MR Tsai CH Role of the TSG101 gene in Epstein-Barr virus late gene transcription J Virol 2007 81 2459 2471 10.1128/JVI.02289-06 17182691 PMC1865947

[B29] Crump CM Yates C Minson T Herpes simplex virus type 1 cytoplasmic envelopment requires functional Vps4 J Virol 2007 81 7380 7387 10.1128/JVI.00222-07 17507493 PMC1933334

[B30] Honeychurch KM Yang G Jordan R Hruby DE The vaccinia virus F13L YPPL motif is required for efficient release of extracellular enveloped virus J Virol 2007 81 7310 7315 10.1128/JVI.00034-07 17475658 PMC1933313

[B31] Kian Chua P Lin MH Shih C Potent inhibition of human Hepatitis B virus replication by a host factor Vps4 Virology 2006 354 1 6 10.1016/j.virol.2006.07.018 16920176

[B32] Lambert C Doring T Prange R Hepatitis B virus maturation is sensitive to functional inhibition of ESCRT-III, Vps4, and gamma 2-adaptin J Virol 2007 81 9050 9060 10.1128/JVI.00479-07 17553870 PMC1951427

[B33] Watanabe T Sorensen EM Naito A Schott M Kim S Ahlquist P Involvement of host cellular multivesicular body functions in hepatitis B virus budding Proc Natl Acad Sci USA 2007 104 10205 10210 10.1073/pnas.0704000104 17551004 PMC1891263

[B34] Chiou CT Hu CC Chen PH Liao CL Lin YL Wang JJ Association of Japanese encephalitis virus NS3 protein with microtubules and tumour susceptibility gene 101 (TSG101) protein J Gen Virol 2003 84 2795 2805 10.1099/vir.0.19201-0 13679614

[B35] Carpp LN Galler R Bonaldo MC Interaction between the yellow fever virus nonstructural protein NS3 and the host protein Alix contributes to the release of infectious particles Microbes Infect 2011 13 85 95 10.1016/j.micinf.2010.10.010 21044891

[B36] Bieniasz PD Late budding domains and host proteins in enveloped virus release Virology 2006 344 55 63 10.1016/j.virol.2005.09.044 16364736

[B37] Demirov DG Freed EO Retrovirus budding Virus Res 2004 106 87 102 10.1016/j.virusres.2004.08.007 15567490

[B38] Hanna SL Pierson TC Sanchez MD Ahmed AA Murtadha MM Doms RW N-linked glycosylation of west nile virus envelope proteins influences particle assembly and infectivity J Virol 2005 79 13262 13274 10.1128/JVI.79.21.13262-13274.2005 16227249 PMC1262570

[B39] Davey NE Van Roey K Weatheritt RJ Toedt G Uyar B Altenberg B Budd A Diella F Dinkel H Gibson TJ Attributes of short linear motifs Molecular bioSystems 2012 8 268 281 10.1039/c1mb05231d 21909575

[B40] Ren S Yang G He Y Wang Y Li Y Chen Z The conservation pattern of short linear motifs is highly correlated with the function of interacting protein domains BMC genomics 2008 9 452 10.1186/1471-2164-9-452 18828911 PMC2576256

[B41] Pornillos O Higginson DS Stray KM Fisher RD Garrus JE Payne M He GP Wang HE Morham SG Sundquist WI HIV Gag mimics the Tsg101-recruiting activity of the human Hrs protein J Cell Biol 2003 162 425 434 10.1083/jcb.200302138 12900394 PMC2172688

[B42] Sayers EW Barrett T Benson DA Bolton E Bryant SH Canese K Chetvernin V Church DM Dicuccio M Federhen S Database resources of the National Center for Biotechnology Information Nucleic Acids Res 2012 40 D13 D25 10.1093/nar/gkr1184 22140104 PMC3245031

[B43] Katoh K Toh H Recent developments in the MAFFT multiple sequence alignment program Brief Bioinform 2008 9 286 298 10.1093/bib/bbn013 18372315

[B44] Waterhouse AM Procter JB Martin DM Clamp M Barton GJ Jalview Version 2–a multiple sequence alignment editor and analysis workbench Bioinformatics 2009 25 1189 1191 10.1093/bioinformatics/btp033 19151095 PMC2672624

[B45] Boratyn GM Schaffer AA Agarwala R Altschul SF Lipman DJ Madden TL Domain enhanced lookup time accelerated BLAST Biol Direct 2012 7 12 10.1186/1745-6150-7-12 22510480 PMC3438057

[B46] Pierson TC Sanchez MD Puffer BA Ahmed AA Geiss BJ Valentine LE Altamura LA Diamond MS Doms RW A rapid and quantitative assay for measuring antibody-mediated neutralization of West Nile virus infection Virology 2006 346 53 65 10.1016/j.virol.2005.10.030 16325883

[B47] Joshi A Garg H Ablan S Freed EO Nagashima K Manjunath N Shankar P Targeting the HIV entry, assembly and release pathways for anti-HIV gene therapy Virology 2011 415 95 106 10.1016/j.virol.2011.03.028 21529874 PMC3107932

[B48] Demirov DG Ono A Orenstein JM Freed EO Overexpression of the N-terminal domain of TSG101 inhibits HIV-1 budding by blocking late domain function Proc Natl Acad Sci USA 2002 99 955 960 10.1073/pnas.032511899 11805336 PMC117412

[B49] Goila-Gaur R Demirov DG Orenstein JM Ono A Freed EO Defects in human immunodeficiency virus budding and endosomal sorting induced by TSG101 overexpression J Virol 2003 77 6507 6519 10.1128/JVI.77.11.6507-6519.2003 12743307 PMC155030

[B50] Bishop N Woodman P TSG101/mammalian VPS23 and mammalian VPS28 interact directly and are recruited to VPS4-induced endosomes J Biol Chem 2001 276 11735 11742 10.1074/jbc.M009863200 11134028

[B51] Joshi A Munshi U Ablan SD Nagashima K Freed EO Functional replacement of a retroviral late domain by ubiquitin fusion Traffic 2008 9 1972 1983 10.1111/j.1600-0854.2008.00817.x 18817521 PMC2763539

[B52] Shehu-Xhilaga M Ablan S Demirov DG Chen C Montelaro RC Freed EO Late domain-dependent inhibition of equine infectious anemia virus budding J Virol 2004 78 724 732 10.1128/JVI.78.2.724-732.2004 14694104 PMC368837

[B53] Lee S Joshi A Nagashima K Freed EO Hurley JH Structural basis for viral late-domain binding to Alix Nat Struct Mol Biol 2007 14 194 199 10.1038/nsmb1203 17277784 PMC2377018

[B54] Munshi UM Kim J Nagashima K Hurley JH Freed EO An Alix fragment potently inhibits HIV-1 budding: characterization of binding to retroviral YPXL late domains J Biol Chem 2007 282 3847 3855 17158451 10.1074/jbc.M607489200

[B55] Schlundt A Sticht J Piotukh K Kosslick D Jahnke N Keller S Schuemann M Krause E Freund C Proline-rich sequence recognition: II. Proteomics analysis of Tsg101 ubiquitin-E2-like variant (UEV) interactions Mol Cell Proteomics 2009 8 2474 2486 10.1074/mcp.M800337-MCP200 19542561 PMC2773715

[B56] Demirov DG Orenstein JM Freed EO The late domain of human immunodeficiency virus type 1 p6 promotes virus release in a cell type-dependent manner J Virol 2002 76 105 117 10.1128/JVI.76.1.105-117.2002 11739676 PMC135729

[B57] Krieger E Koraimann G Vriend G Increasing the precision of comparative models with YASARA NOVA–a self-parameterizing force field Proteins 2002 47 393 402 10.1002/prot.10104 11948792

[B58] Nybakken GE Nelson CA Chen BR Diamond MS Fremont DH Crystal structure of the West Nile virus envelope glycoprotein J Virol 2006 80 11467 11474 10.1128/JVI.01125-06 16987985 PMC1642602

[B59] Kaufmann B Vogt MR Goudsmit J Holdaway HA Aksyuk AA Chipman PR Kuhn RJ Diamond MS Rossmann MG Neutralization of West Nile virus by cross-linking of its surface proteins with Fab fragments of the human monoclonal antibody CR4354 Proc Natl Acad Sci USA 2010 107 18950 18955 10.1073/pnas.1011036107 20956322 PMC2973864

[B60] Pawliczek T Crump CM Herpes simplex virus type 1 production requires a functional ESCRT-III complex but is independent of TSG101 and ALIX expression J Virol 2009 83 11254 11264 10.1128/JVI.00574-09 19692479 PMC2772754

[B61] Irie T Harty RN L-domain flanking sequences are important for host interactions and efficient budding of vesicular stomatitis virus recombinants J Virol 2005 79 12617 12622 10.1128/JVI.79.20.12617-12622.2005 16188963 PMC1235845

[B62] Irie T Licata JM Jayakar HR Whitt MA Bell P Harty RN Functional analysis of late-budding domain activity associated with the PSAP motif within the vesicular stomatitis virus M protein J Virol 2004 78 7823 7827 10.1128/JVI.78.14.7823-7827.2004 15220457 PMC434086

[B63] Dowlatshahi DP Sandrin V Vivona S Shaler TA Kaiser SE Melandri F Sundquist WI Kopito RR ALIX is a Lys63-specific polyubiquitin binding protein that functions in retrovirus budding Dev Cell 2012 23 1247 1254 10.1016/j.devcel.2012.10.023 23201121 PMC3522770

[B64] Keren-Kaplan T Attali I Estrin M Kuo LS Farkash E Jerabek-Willemsen M Blutraich N Artzi S Peri A Freed EO Structure-based in silico identification of ubiquitin-binding domains provides insights into the ALIX-V:ubiquitin complex and retrovirus budding The EMBO journal 2013 32 538 551 10.1038/emboj.2013.4 23361315 PMC3579145

[B65] Ko A Lee EW Yeh JY Yang MR Oh W Moon JS Song J MKRN1 induces degradation of West Nile virus capsid protein by functioning as an E3 ligase J Virol 2010 84 426 436 10.1128/JVI.00725-09 19846531 PMC2798448

[B66] Martin-Serrano J The role of ubiquitin in retroviral egress Traffic 2007 8 1297 1303 10.1111/j.1600-0854.2007.00609.x 17645437

[B67] Ng ML Howe J Sreenivasan V Mulders JJ Flavivirus West Nile (Sarafend) egress at the plasma membrane Arch Virol 1994 137 303 313 10.1007/BF01309477 7944952

[B68] Seligman SJ Bucher DJ The importance of being outer: consequences of the distinction between the outer and inner surfaces of flavivirus glycoprotein E Trends Microbiol 2003 11 108 110 10.1016/S0966-842X(03)00005-2 12648938

[B69] Danecek P Lu W Schein CH PCP consensus sequences of flaviviruses: correlating variance with vector competence and disease phenotype Journal of molecular biology 2010 396 550 563 10.1016/j.jmb.2009.11.070 19969003 PMC2822003

[B70] Adachi A Gendelman HE Koenig S Folks T Willey R Rabson A Martin MA Production of acquired immunodeficiency syndrome-associated retrovirus in human and nonhuman cells transfected with an infectious molecular clone J Virol 1986 59 284 291 3016298 10.1128/jvi.59.2.284-291.1986 PMC253077

[B71] Patnaik A Chau V Li F Montelaro RC Wills JW Budding of equine infectious anemia virus is insensitive to proteasome inhibitors J Virol 2002 76 2641 2647 10.1128/JVI.76.6.2641-2647.2002 11861830 PMC135976

[B72] Freed EO Orenstein JM Buckler-White AJ Martin MA Single amino acid changes in the human immunodeficiency virus type 1 matrix protein block virus particle production J Virol 1994 68 5311 5320 8035531 10.1128/jvi.68.8.5311-5320.1994 PMC236481

